# From hypercortisolism to remission: impact of Cushing’s disease on eating patterns

**DOI:** 10.3389/fendo.2026.1726118

**Published:** 2026-02-02

**Authors:** Merve Korkmaz Yilmaz, Huseyin Sehit Burhan, Sebnem Burhan, Mutlu Niyazoglu, Esra Hatipoglu

**Affiliations:** 1 Department of Endocrinology, University of Health Sciences, Basaksehir Cam and Sakura City Hospital, Istanbul, Türkiye; 2 Department of Psychiatry, University of Health Sciences, Basaksehir Cam and Sakura City Hospital, Istanbul, Türkiye; 3 University of Health Sciences, Pituitary Disorders Research Center, Istanbul, Türkiye

**Keywords:** Cushing’s disease, disease activity, eating behavior, emotional regulation, hypercortisolism, mindfulness, remission

## Abstract

**Background:**

Cushing’s disease (CD) is characterized by chronic hypercortisolism and is associated with persistent metabolic, psychological, and neurocognitive disturbances. While metabolic consequences are well described, the impact of disease activity and remission on eating behavior remains insufficiently explored.

**Objective:**

This study aimed to compare multidimensional eating behavior patterns among patients with active Cushing’s disease, patients in biochemical remission, and healthy controls, and to examine their associations with cortisol biomarkers.

**Methods:**

In this cross-sectional study, 74 participants were enrolled, including patients with active CD (n = 21), patients in remission (n = 32), and age-, sex-, and BMI-matched healthy controls (n = 21). Eating behavior was assessed using validated questionnaires: the Night Eating Questionnaire (NEQ), Emotional Appetite Questionnaire (EMAQ), Three-Factor Eating Questionnaire–Revised 18 (TFEQ-R18), Mindful Eating Questionnaire (MEQ), and Dutch Eating Behavior Questionnaire (DEBQ). Group differences were analyzed using Kruskal–Wallis tests followed by Dunn’s *post-hoc* comparisons with Bonferroni correction. Associations between eating behavior scores and cortisol parameters were evaluated using Spearman correlation analysis.

**Results:**

Active Cushing’s disease was associated with higher night eating (NEQ; p < 0.001) and greater emotional and situational eating compared with remission and healthy controls (EMAQ total; p < 0.001). Positive emotion and situation subscales were higher in active disease (both p = 0.008), whereas negative total scores differed significantly only between active disease and healthy controls (p = 0.014). Mindful eating was reduced in both patient groups versus controls (MEQ total; p < 0.001), with active disease showing higher disinhibition (p = 0.002), greater interference (p < 0.001), and lower conscious nutrition (p = 0.016). Remission patients demonstrated partial but incomplete behavioral recovery. Late-night salivary cortisol correlated with MEQ interference (r = 0.5, p = 0.01), and cortisol levels after the 1-mg DST correlated with DEBQ emotional eating (r = 0.5, p = 0.01).

**Conclusion:**

Cushing’s disease is associated with marked alterations in eating behavior, particularly during active disease, including increased night eating, emotional susceptibility, and reduced mindful regulation. Although partial improvement occurs after remission, residual behavioral disturbances persist. These findings underscore the importance of integrating behavioral assessment into the long-term management of Cushing’s disease.

## 1 Introduction

Cushing’s syndrome (CS) is a rare but complex endocrine disorder resulting from chronic exposure to excess glucocorticoids, most frequently due to adrenocorticotropic hormone (ACTH)-secreting pituitary adenomas or adrenal tumors (1). A broad range of physical, metabolic, and psychiatric manifestations characterizes the clinical phenotype. Clinically, patients typically present with central obesity, muscle atrophy, hypertension, diabetes mellitus, and dyslipidemia (2). Characteristic changes in body appearance, including facial rounding (“moon face”), dorsocervical fat pad (“buffalo hump”), and abdominal fat accumulation, contribute not only to somatic morbidity but also to significant psychosocial distress (3).

Beyond metabolic complications, CS is associated with profound neuropsychiatric sequelae. Depression, anxiety, irritability, cognitive impairment, and sleep disturbances are frequently observed, and many of these symptoms persist even after biochemical remission (4, 5). These findings suggest that hypercortisolism exerts long-lasting effects on brain structure and function. Neuroimaging studies have demonstrated alterations in the hippocampus, amygdala, and prefrontal cortex—regions that play a crucial role in emotional regulation, memory, and impulse control (6, 7).

Eating behavior is one domain that is particularly sensitive to such neuroendocrine disruptions but has been underexplored in the context of CS. In the general population, maladaptive eating patterns—such as emotional eating, night eating, and reduced mindful eating—are strongly linked to obesity, metabolic syndrome, and mood disorders (8, 9). Dysregulated eating has been mechanistically connected to hyperactivation of the hypothalamic–pituitary–adrenal (HPA) axis, dysregulation of appetite-related hormones such as leptin and ghrelin, and alterations in dopaminergic reward pathways (10, 11). Given that CS represents a striking human model of chronic HPA axis hyperactivity, studying eating behaviors in this population offers unique insights into the interface between endocrine dysregulation and maladaptive food intake.

Several pathways may underlie disordered eating in CS. Cortisol directly influences appetite regulation by enhancing ghrelin activity and impairing leptin sensitivity, thereby promoting hyperphagia and preference for high-calorie foods (12, 13). Psychiatric comorbidities—particularly depression, anxiety, and reduced self-esteem—further predispose patients to emotional eating as a maladaptive coping strategy (14). In addition, changes in body image due to disease-specific physical features may exacerbate vulnerability to disordered eating (15). The frequent coexistence of psychiatric disorders, including major depression and generalized anxiety disorder, reinforces these vulnerabilities and contributes to a multidimensional risk profile (16).

Although metabolic consequences of CS have been extensively studied, there is a paucity of data specifically addressing its association with eating behavior using validated psychometric tools. Preliminary findings suggest that patients with active CS exhibit increased appetite, emotional dysregulation, and reduced cognitive restraint (17). However, whether these abnormalities fully resolve following remission remains uncertain. Some evidence indicates that maladaptive eating patterns may persist despite biochemical cure, raising questions about reversibility and their contribution to long-term weight gain, cardiometabolic risk, and impaired quality of life (18).

Recent advances in behavioral endocrinology emphasize integrating psychological constructs, such as emotional eating, cognitive restraint, disinhibition, mindful eating, and night eating, into the evaluation of endocrine disorders (19). Validated questionnaires—including the Night Eating Questionnaire (NEQ), Emotional Appetite Questionnaire (EMAQ), Three-Factor Eating Questionnaire (TFEQ-R18), Mindful Eating Questionnaire (MEQ), and Dutch Eating Behavior Questionnaire (DEBQ)—allow multidimensional assessment of eating patterns. Yet, they have rarely been applied in CS populations (20, 21).

Therefore, the present study aimed to systematically compare eating behavior patterns among patients with active Cushing’s disease (CD), patients in remission, and healthy controls, and to examine their associations with cortisol biomarkers. By identifying behavioral phenotypes associated with disease activity, this study aims to provide novel insights into the psychosocial burden of CD and inform comprehensive management strategies that integrate endocrinological, psychiatric, and nutritional perspectives.

## 2 Method

### 2.1 Study population and procedures

This cross-sectional study included a total of 74 participants, divided into three groups: patients with active Cushing’s disease (n = 21), patients with Cushing’s disease in biochemical remission following treatment (n = 32), and age-, sex-, and BMI-matched healthy controls (n = 21). All patients were recruited from the Endocrinology Outpatient Clinic of Basaksehir Cam and Sakura City Hospital between January 2021 and June 2024.

All patients were diagnosed with ACTH-dependent Cushing’s disease. Hypercortisolism was established based on elevated urinary free cortisol, late-night salivary and serum cortisol levels, and lack of suppression after the 1-mg overnight dexamethasone suppression test (DST). To determine the etiology of hypercortisolism, plasma ACTH levels were assessed, and additional pituitary–adrenal function tests were performed as clinically indicated, including dynamic testing and pituitary imaging, leading to the diagnosis of pituitary-dependent disease. Non-neoplastic causes of hypercortisolism (pseudo-Cushing states) were systematically excluded. All diagnoses were subsequently confirmed by histopathological examination following transsphenoidal pituitary surgery.

The diagnosis of active Cushing’s disease and confirmation of remission status were established according to standard biochemical criteria, including 24-hour urinary free cortisol (UFC), late-night salivary cortisol (LNSC), and the 1-mg overnight DST. Remission was defined biochemically as normalization of UFC and LNSC and adequate cortisol suppression after the 1-mg DST (<1.8 mcg/dL). In addition, the absence or regression of Cushingoid clinical features was required to support remission status.

A comprehensive clinical and sociodemographic evaluation was conducted for all participants. Collected variables included age, sex, level of education, monthly income, overall socioeconomic status, and the presence of hypertension and diabetes mellitus. Disease-related parameters recorded for patients included disease duration, pituitary adenoma size at diagnosis, body mass index, and fasting plasma glucose.

The healthy control group consisted of individuals with no known endocrine, psychiatric, or metabolic disorders and with biochemically confirmed normal hypothalamic–pituitary–adrenal axis function, as evidenced by adequate cortisol suppression following the 1-mg DST.

Eligibility criteria required participants to be between 18 and 70 years of age. Exclusion criteria for all groups included pregnancy, use of psychiatric medications, or cognitive impairment that could interfere with the completion of self-report questionnaires. The study was conducted in accordance with the Declaration of Helsinki. Written informed consent was obtained from all participants, and the study protocol was approved by the Institutional Ethics Committee of Basaksehir Cam and Sakura City Hospital (approval number: 250/25.06.2025).

### 2.2 Hormonal assessments

Plasma cortisol and adrenocorticotropic hormone (ACTH) concentrations were measured using electrochemiluminescence immunoassay (ECLIA) methods (Elecsys Cortisol II and ACTH kits, Roche Diagnostics) on Cobas analyzers. Late-night salivary cortisol (LNSC) was measured by ECLIA using the Elecsys Cortisol II kit (Roche Diagnostics), with an assay-specific upper limit of normal of 0.41 mcg/dL.

Twenty-four–hour urinary free cortisol (UFC) was quantified using liquid chromatography–tandem mass spectrometry (LC–MS/MS) (Agilent 6460 Triple Quadrupole system), with an upper limit of normal of 45 mcg/24 h.

In our center, serum cortisol was additionally measured at 23:00 h while patients were awake; bedtime serum cortisol values exceeding 7.5 mcg/dL were considered supportive of hypercortisolism.

For patients with Cushing’s disease, biochemical parameters related to hypercortisolism—except for dexamethasone suppression tests—were obtained on two separate days, and mean values were used for analysis.

### 2.3 Measures

Eating behavior was assessed using a comprehensive set of validated psychometric instruments, each targeting distinct cognitive, emotional, and behavioral domains related to food intake (**Table 1**).

**Table 1:** Summary of eating behavior questionnaires and their behavioral domains.

Questionnaire	Main behavioral domains	Subscales	Interpretation of higher scores	Reference
Night Eating Questionnaire (NEQ)	Nocturnal eating patterns and circadian rhythm disruption	Evening hyperphagia, nocturnal awakenings to eat, morning anorexia, mood/sleep disturbances	Greater night eating severity and disrupted eating-sleep cycle	28 29
Emotional Appetite Questionnaire (EMAQ)	Emotional and situational influences on food intake	Positive emotions (PE), Negative emotions (NE), Positive situations (PS), Negative situations (NS), Positive total (PTS), Negative total (NTS)	Higher emotional reactivity to food cues under affective or contextual conditions	30 31
Three-Factor Eating Questionnaire-Revised 18 (TFEQ-R18)	Cognitive and emotional control of eating	Uncontrolled eating, Emotional eating, Cognitive restraint	Greater uncontrolled and emotional eating tendencies; higher restraint reflects increased dietary control	32 33
Mindful Eating Questionnaire (MEQ)	Awareness, attention, and self-regulation during eating	Disinhibition, Emotional eating, Eating control, Mindfulness, Eating discipline, Conscious nutrition, Interference	Higher scores indicate greater mindfulness and self-regulation; lower scores reflect distraction and emotional influence	21 34
Dutch Eating Behavior Questionnaire (DEBQ)	Cognitive, emotional, and external eating styles	Restrained eating, Emotional eating, External eating	Higher scores indicate a greater tendency toward the respective eating style	35 36

All instruments were administered in validated Turkish versions through structured face-to-face interviews. Higher scores in each subscale reflect greater expression of the corresponding behavioral dimension.

*Night Eating Questionnaire (NEQ):* Evaluates nocturnal eating patterns, including evening hyperphagia, nocturnal awakenings to eat, morning anorexia, and mood or sleep disturbances related to eating. Higher scores indicate greater severity of night eating symptoms.

*Emotional Appetite Questionnaire (EMAQ):* This questionnaire evaluates the impact of both emotional states and situational factors on eating, with subscales assessing positive and negative emotions, as well as positive and negative situational triggers.

*Three-Factor Eating Questionnaire – Revised 18 (TFEQ-R18):* This questionnaire captures three core domains of eating behavior: uncontrolled eating, emotional eating, and cognitive restraint.

*Mindful Eating Questionnaire (MEQ):* The MEQ was employed to evaluate attentional awareness and self-regulation during eating. In this study, seven specific subscales were analyzed: disinhibition, emotional eating, eating control, mindfulness, eating discipline, conscious nutrition, and interference. These domains collectively capture the extent to which individuals are aware of their eating behavior, regulate food intake, and resist internal or external triggers. Higher scores indicate greater mindfulness and healthier eating patterns.

*Dutch Eating Behavior Questionnaire (DEBQ):* Evaluates restrained, emotional, and external eating styles, widely applied in studies of eating disorders and obesity.

All questionnaires were administered in their Turkish-validated versions through structured, face-to-face interviews conducted by trained researchers to ensure accuracy and reliability of responses.

### 2.4 Statistical analysis

All statistical analyses were performed using IBM SPSS Statistics for Windows, Version 25.0 (IBM Corp., Armonk, NY, USA). Descriptive statistics are presented as frequencies and percentages for categorical variables, and as medians with interquartile ranges (IQR) or means ± standard deviations (SD) for continuous variables, as appropriate.

Normality of data distribution was assessed using the Kolmogorov–Smirnov test. Because most continuous variables deviated from normality (p < 0.05), nonparametric statistical methods were used. Comparisons among more than two independent groups were conducted using the Kruskal–Wallis test. When a significant overall difference was detected, pairwise comparisons were performed using Dunn’s *post-hoc* test with Bonferroni correction, with an adjusted significance threshold of p < 0.017.

Comparisons between two independent groups were performed using the Mann–Whitney U test only when analyses were limited to two groups. Categorical variables were analyzed using Pearson’s chi-square test or Fisher’s exact test, depending on expected cell frequencies.

Associations between eating behavior questionnaire scores and biochemical cortisol parameters—including 24-hour urinary free cortisol (UFC), late-night salivary cortisol (LNSC), and cortisol levels after the 1-mg dexamethasone suppression test (DST)—were assessed using Spearman’s rank correlation coefficient. All statistical tests were two-tailed, and a p-value < 0.05 was considered statistically significant unless otherwise specified.

## 3 Results

### 3.1 Sociodemographic and clinical characteristics

A total of 74 participants were included in the study: 21 with active Cushing’s disease, 32 in remission, and 21 healthy controls. The groups were similar with respect to age, gender distribution, education level, presence of hypertension (HT), diabetes mellitus (DM), and income distribution (all p > 0.05) (**Table 2**).

**Table 2:** Comparison of sociodemographic and clinical characteristics across study groups.

Variables	Active CD (n=21)	Remission CD (n=32)	Healthy controls (n=21)	P - value
Age (yrs)				
Mean ± SD	51,0±7.7	47,5**±**10.4	50,0±10.4	0.7^a^
Gender				
Female, n(%)	19 (90)	21 (66)	18 (86)	0.09^c^
Male, n(%)	2 (10)	11 (34)	3 (14)	
Education				
Primary school, n(%)	5 (24)	11 (34)	5 (24)	0.4^b^
High school, n(%)	7 (33)	9 (28)	3 (14)	
University, n(%)	9 (43)	12 (38)	13 (62)	
Income				
Low income (<34,000 TL/month), n(%)	2 (10)	8 (25)	5 (24)	0.2^b^
Middle income (34,000–68,000 TL/month), n(%)	7 (33)	5 (16)	8 (38)	
High income (>68,000 TL/month), n(%)	12 (57)	19 (59)	8 (38)	
Hypertension				
No, n(%)	11 (52)	19 (59)	11 (52)	0.8^b^
Yes, n(%)	10 (48)	13 (41)	10 (48)	
Diabetes mellitus				
No, n(%)	11 (52)	23 (72)	11 (52)	0.2^b^
Yes, n(%)	10 (48)	9 (28)	10 (48)	
Pituitary tumor size (mm)				
Median (IQR)	7,0 (5-15)	11,0 (9.5-21)		**0.04^d^**
** Duration of disease (yrs)**				
Median (IQR)	2,0 (1-3)	2,0 (1-3)		0.5^d^
Fasting blood plasma glucose (mcg/dl)				
Median (IQR)	100,0 (90-110)	93,0 (90-102)	90,0 (90-94)	**0.04^a^**
** BMI (kg/m²)**				
Median (IQR)	33,0 (27-37)	32,0 (29-35)	30,0 (28-35)	0.4^a^
24 h-urinary free cortisole (UFC)				
Median (IQR)	73,0 (53-117)	16,0 (11-21)		**<0.001^d^**
Late-night salivary cortisol (LNSC)				
Median (IQR)	0,44 (0,2-0.6)	0,07 (0,06-0,13)		**<0.001^d^**
1-mg dexamethasone suppression test (1-mg DST)				
Median (IQR)	6,8 (4.2-9.6)	0,8 (0,6-1,2)	0,5 (0,5-0,6)	**<0.001^a^**

a: Kruskal–Wallis test; b: Pearson’s Chi-square test; c: Fisher’s exact test; d: Mann–Whitney U test. TL: Turkish lira. p < 0.05 was considered statistically significant. Bold values indicate statistically significant p-values (p < 0.05).

Regarding disease-related clinical parameters, pituitary tumor size was significantly smaller in the active group compared with the remission group (p = 0.04). Fasting plasma glucose levels were also significantly higher in the active group relative to the other groups (p = 0.04). No significant difference in body mass index (BMI) was observed across groups (p = 0.4).

Median 24-hour urinary free cortisol (UFC), late-night salivary cortisol (LNSC), and the 1-mg dexamethasone suppression test (DST) values were all significantly elevated in the active group compared with remission patients and healthy controls (all p < 0.001), underscoring their sensitivity in differentiating disease activity.

### 3.2 Comparison of eating behavior measures

When comparing all three groups (**Table 3**), differences in eating behavior measures were predominantly driven by higher scores in patients with active Cushing’s disease, as confirmed by Dunn’s *post hoc* analyses with Bonferroni correction.

**Table 3:** Comparison of scale and subscale scores across study groups.

Variables	Active CD (n=21)	Remission CD (n=32)	Healthy Controls (n=21)	P - value	Dunn post-hoc (Bonferroni)
Night Eating Questionnaire (NEQ)	22,1±2,9	13,8±4,5	11,3±4,1	<0.001	A>R**; A>H**
The emotional appetite questionnaire (EMAQ) -Total (NTS+PTS)	105.1 ± 21.8	85.8 ± 29.2	81.9 ± 17.4	<0.001	A>R**; A>H**
• EMAQ-NE	43,1±10,9	36,6±14,0	31,1±9,0	0.001	A>H**
• EMAQ-PE	30,5±8,6	24,7±10,4	24,7±6,6	0.008	A>R*; A>H*
• EMAQ-NS	16,9±8,0	13,3±6,9	13,2±5,3	0.185	–
• EMAQ-PS	16,5±5,3	12,2±5,8	12,1±2,6	0.008	A>R*; A>H*
• EMAQ-NTS	58,1±14,9	48,9±18,3	45,1±13,4	0.014	A>H*
• EMAQ-PTS	47±12,7	36,9±15,3	36,7±7,6	0.005	A>R*; A>H*
The Three-Factor Eating Questionnaire-R18 (TFEQ-R18)	47,6±13,0	39,4±5,7	28,6±2,9	<0.001	A>H**; R>H**
• Uncontrolled eating	21,9±10,4	16,3±4,6	10,8±1,7	<0.001	A>H**; R>H**
• Cognitive Restraint	17,9±2,9	17,89±5,2	13,6±2,7	0.001	A>H**; R>H**
• Emotional eating	7,8±3,95	5,3±2,8	4,1±1,8	0.004	A > H*
Mindful Eating Questionnaire (MEQ)	73,6±12,9	80,3±11,0	92,9±9,4	<0.001	H>A**; H>R**
• Disinhibition	15±4,6	10,6±3,5	9,3±3,6	0.002	A>R*; A>H**
• Emotional eating	10,5±2,2	10,3±4,3	13,8±3,7	0.004	H > A*; H > R*
• Eating control	9,0±2,1	8,9±1,9	10,1±2,6	0.069	–
• Mindfulness	16,7±5,4	17,7±3,3	19,1±4,5	0.271	–
• Eating discipline	11,9±5,4	14,3±3,6	15,4±3,7	0.072	–
• Conscious nutrition	12,8±2,6	14,7±3,2	14,9±2,2	0.016	H>A*
• Interference	4,6±1,6	3,9±1,4	2,8±1,0	<0.001	A>R*; A>H**
Dutch Eating Behavior Questionnaire (DEBQ)	87,9±20,8	80,3±16,5	75,7±11,1	0.186	–
• Emotional eating	29,43±14,43	26,84±12,21	25,81±2,32	0.895	–
• Restrained eating	30,67±3,65	29,22±7,48	25,76±9,43	0.142	–
• External eating	24,86±5,1	24,25±6,18	24,14±8,36	0.870	–

Data are presented as mean ± SD.

Group differences were assessed using the Kruskal–Wallis test. When significant, pairwise comparisons were performed using Dunn’s post-hoc test with Bonferroni correction (adjusted significance level p < 0.017).

CD, Cushing’s disease; A, active; R, remission; H, healthy controls.

*p < 0.017, **p < 0.001.

- Night Eating Questionnaire (NEQ): NEQ total scores were significantly higher in the active disease group than in remission patients and healthy controls (p < 0.001 for both comparisons).
- Emotional Appetite Questionnaire (EMAQ): EMAQ total scores (NTS + PTS) were higher in the active group than in remission patients and healthy controls (p < 0.001 for both comparisons). Among subscales, EMAQ negative emotion scores were higher in the active group compared with healthy controls (p < 0.001), whereas positive emotion and positive situation scores were elevated in the active group relative to both remission patients and healthy controls (p = 0.008 for both). The negative total score differed only between the active group and healthy controls (p = 0.014), whereas the positive total score was higher in the active group than in both comparison groups (p = 0.005).
- Three-Factor Eating Questionnaire–Revised 18 (TFEQ-R18): Total TFEQ-R18 scores were higher in both active and remission groups compared with healthy controls (p < 0.001 for both). Uncontrolled eating and cognitive restraint were similarly elevated across patient groups (p < 0.001), whereas emotional eating was higher in the active group than in healthy controls (p = 0.004).
- Mindful Eating Questionnaire (MEQ): MEQ total scores were higher in healthy controls than in both active and remission patients (p < 0.001 for both comparisons), indicating reduced mindful eating in disease states. Among the MEQ subscales, disinhibition was higher in the active group than in remission patients (p = 0.002) and healthy controls (p < 0.001), whereas emotional eating and conscious nutrition scores were higher in healthy controls than in patient groups (p = 0.004 and p = 0.016, respectively). Interference scores were elevated in the active group compared with remission patients (p < 0.017) and healthy controls (p < 0.001). Other MEQ subscales showed no significant group differences.
- Dutch Eating Behavior Questionnaire (DEBQ): No significant *post-hoc* differences were observed between groups for total or subscale scores of the DEBQ.

### 3.3 Correlations between cortisol markers and eating behavior in the active group

Within the active CD group, correlation analyses revealed specific associations between eating behavior patterns and clinical or biochemical parameters.

**Figure 1:**
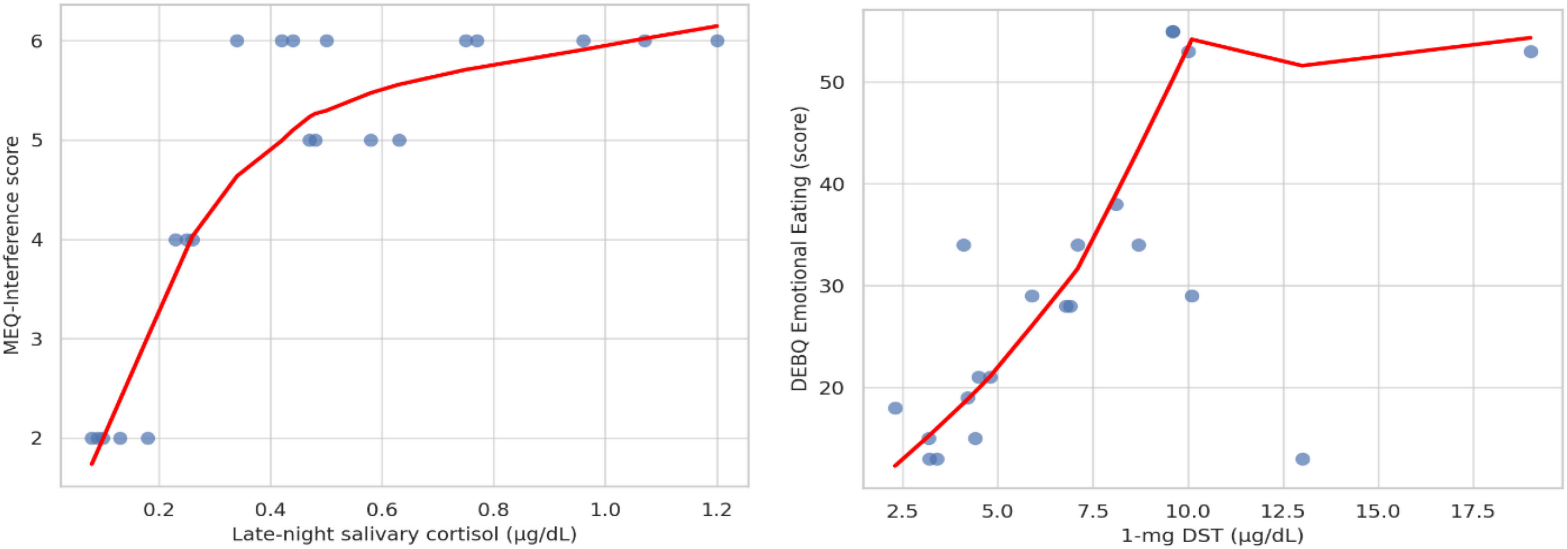
Associations between cortisol biomarkers and eating behavior domains: LNSC vs. MEQ-Interference; 1-mg DST cortisol vs. DEBQ-Emotional Eating. Both correlations were significant, showing positive associations between late-night salivary cortisol (LNSC) and MEQ-Interference scores (r = 0.53, p = 0.01), and between 1-mg DST cortisol levels and DEBQ-Emotional Eating scores (r = 0.51, p = 0.01).

- TFEQ-R18: The total score correlated positively with age (r = 0.36, p = 0.04). Cognitive restraint also showed a positive correlation with age (r = 0.34, p = 0.04).
- MEQ: The mindfulness subscale correlated positively with age (r = 0.34, p = 0.04) and negatively with BMI (r = –0.46, p = 0.03). In addition, the interference subscale demonstrated a significant positive correlation with late-night salivary cortisol (LNSC) (r = 0.53, p = 0.01) (**Figure 1**).
- DEBQ: The emotional eating subscale correlated positively with 1-mg dexamethasone suppression test (DST) values (r = 0.51, p = 0.01) (**Figure 1**).
- No other significant correlations were found between EMAQ subscales or additional MEQ domains and clinical or biochemical variables (all p > 0.05).

Although several correlations did not reach statistical significance, some variables demonstrated a trend toward significance (0.05 < p < 0.1). Specifically, a positive tendency was observed between 1 mg-DST levels and EMAQ-NTS (r = 0.4, p = 0.06) as well as EMAQ-NE (r = 0.39, p = 0.07).

## 4 Discussion

In this study, we demonstrated that patients with active Cushing’s disease exhibit a distinct pattern of maladaptive eating behaviors compared with both patients in remission and healthy controls. Active disease was associated with higher levels of night eating and emotional eating, as well as greater susceptibility to emotional and situational triggers of food intake, as reflected by elevated EMAQ subscale scores. In addition, impairments in mindful eating—particularly in domains related to attentional interference and conscious nutrition—suggest reduced awareness and self-regulation during eating. Although patients in remission showed partial behavioral improvement, their eating behavior profiles did not fully normalize, indicating that some disturbances persist beyond biochemical remission. Correlation analyses further demonstrated associations between cortisol biomarkers and maladaptive eating dimensions, supporting a link between hypercortisolism and dysregulated cognitive–emotional control of appetite.

Our findings reinforce and extend previous evidence on the behavioral consequences of chronic cortisol excess. Geer et al. reported increased food cravings—especially for sweet and salty foods—in patients with active Cushing’s disease compared with those in remission, suggesting a direct relationship between cortisol exposure and hedonic appetite (22). Similarly, Moeller et al. demonstrated altered reward sensitivity during food-choice tasks in active disease, with partial normalization following biochemical cure (23). The elevated NEQ and EMAQ scores observed in our cohort are consistent with these findings and suggest that emotional and night-eating tendencies may represent a persistent psychological footprint of hypercortisolism. These patterns may reflect a sustained imbalance between limbic reward sensitivity and prefrontal inhibitory control, even after biochemical remission, indicating incomplete neurocognitive recovery.

While prior studies have primarily emphasized negative affect and stress-related cues as drivers of disordered eating in Cushing’s disease (22, 23), our results extend this concept by demonstrating that positive emotional contexts may also contribute to maladaptive eating. Elevated EMAQ positive emotion and situation scores suggest that cortisol dysregulation may amplify hedonic responsiveness not only under stress but also in rewarding environments, indicating a broader disruption of reward processing and self-regulatory control. The observed discrepancy between significant EMAQ total negative scores and non-significant negative subscale scores likely reflects greater statistical power of the composite score and heterogeneous responses to negative emotional and situational triggers.

The persistence of behavioral abnormalities in remission parallels previous reports of incomplete recovery in affective and cognitive domains. Studies by Ragnarsson et al. and Wagenmakers et al. demonstrated ongoing fatigue, depressive symptoms, and reduced quality of life despite biochemical remission (6, 24). Structural neuroimaging studies have further shown long-lasting alterations in hippocampal and prefrontal cortical regions following chronic hypercortisolism (25, 26). In line with these observations, the positive correlation between late-night salivary cortisol and mindful eating interference in our study suggests that sustained cortisol exposure may impair attentional control during eating, reflecting persistent HPA axis–related effects on cognitive regulation.

We also observed a physiological association between emotional eating and cortisol suppression. Higher DEBQ emotional eating scores correlated with elevated cortisol levels following the 1-mg dexamethasone suppression test, indicating that impaired negative feedback of the HPA axis may enhance emotional reactivity toward food cues. This finding aligns with evidence linking disrupted cortisol feedback to increased limbic system responsivity and emotional overeating (27). Together, these results suggest that hypercortisolism simultaneously weakens cognitive control mechanisms while strengthening affective drives, creating a behavioral phenotype characterized by reduced mindfulness and heightened emotional eating.

From a mechanistic perspective, chronic cortisol excess influences appetite regulation through both peripheral and central pathways. Elevated cortisol promotes ghrelin secretion, reduces leptin sensitivity, and enhances dopaminergic signaling in reward-related brain regions, thereby increasing hunger, delaying satiety, and amplifying preference for palatable foods (11). Together, these endocrine and neural effects may create a feed-forward loop in which cortisol-driven reward sensitivity reinforces hedonic drive while simultaneously weakening inhibitory control over eating behavior, contributing to an imbalance between emotional reactivity and mindful regulation of food intake.

Beyond biological mechanisms, psychosocial factors may also contribute to disordered eating in Cushing’s disease. Disease-specific physical changes and altered body image can increase emotional distress and reduce self-regulatory capacity, thereby promoting compensatory eating behaviors. These psychosocial influences are likely to interact with chronic cortisol excess, collectively reinforcing maladaptive eating patterns.

Clinically, these findings underscore the importance of integrating behavioral assessment into the long-term management of Cushing’s disease. Screening tools such as the NEQ, EMAQ, and MEQ may help identify patients at risk for persistent maladaptive eating patterns during remission. While traditional management strategies emphasize biochemical control, our results suggest that behavioral and psychological sequelae remain relevant and may benefit from targeted interventions. Mindfulness-based approaches and cognitive–behavioral strategies addressing emotional eating could support recovery of self-regulatory control and reduce the risk of long-term weight regain and metabolic deterioration. Early integration of structured behavioral interventions during remission may therefore represent an opportunity to facilitate neurobehavioral rehabilitation alongside endocrine recovery.

The strengths of this study include the comprehensive evaluation of eating behavior using multiple validated instruments and the inclusion of both active and remission patients, alongside matched healthy controls, which allows differentiation between acute and persistent behavioral effects. Nevertheless, several limitations should be acknowledged. The relatively small sample size may limit statistical power, and the cross-sectional design precludes causal inference. Additionally, reliance on self-report questionnaires may introduce recall or social desirability bias, and potential confounders such as psychiatric comorbidities and sleep disturbances were not systematically assessed.

In summary, this study provides a multidimensional characterization of eating behavior in Cushing’s disease. It demonstrates that chronic hypercortisolism is associated with enduring alterations in emotional, cognitive, and mindful aspects of food intake. Although behavioral disturbances improve following biochemical remission, they do not fully resolve, indicating that cortisol excess may leave lasting effects on appetite regulation and self-regulatory control. These findings highlight the need to consider behavioral dimensions in long-term follow-up, even after hormonal normalization.

## 5 Conclusion

Our findings show that Cushing’s disease, particularly during active disease, is associated with significant disruptions in eating behavior, including heightened night eating, increased emotional susceptibility, and reduced mindful awareness. While partial behavioral recovery occurs after remission, residual vulnerabilities persist, underscoring the long-lasting impact of chronic hypercortisolism. Integrating psychological and nutritional perspectives into endocrine care may support more complete recovery and improve long-term metabolic and quality-of-life outcomes.

## Data Availability

The original contributions presented in the study are included in the article/supplementary material. Further inquiries can be directed to the corresponding author/s.
